# Assessment of the Nutraceutical Effects of Oleuropein and the Cytotoxic Effects of Adriamycin, When Administered Alone and in Combination, in MG-63 Human Osteosarcoma Cells

**DOI:** 10.3390/nu13020354

**Published:** 2021-01-25

**Authors:** Katerina Gioti, Anastasia Papachristodoulou, Dimitra Benaki, Nektarios Aligiannis, Alexios-Leandros Skaltsounis, Emmanuel Mikros, Roxane Tenta

**Affiliations:** 1 Department of Nutrition and Dietetics, School of Health Science and Education, Harokopio University, Kallithea, 17671 Athens, Greece; catherine_geo@yahoo.com (K.G.); anpapac@pharm.uoa.gr (A.P.); 2 Department of Pharmacy, Section of Pharmaceutical Chemistry, National and Kapodostrian University of Athens, Panepistimiopolis, Zografou, 15771 Athens, Greece; dbenaki@pharm.uoa.gr (D.B.); mikros@pharm.uoa.gr (E.M.); 3 Department of Pharmacy, Section of Pharmacognosy and Natural Products Chemistry, National and Kapodistrian University of Athens, Panepistimiopolis, Zografou, 15771 Athens, Greece; aligiannis@pharm.uoa.gr (N.A.); skaltsounis@pharm.uoa.gr (A.-L.S.)

**Keywords:** oleuropein, adriamycin, osteosarcoma

## Abstract

Oleuropein (OLEU) is the most distinguished phenolic compound found in olive fruit and the leaves of *Olea europaea* L., with several pharmacological properties, including anti-cancer actions. Adriamycin (ADR) is an anthracycline widely used as a chemotherapeutic agent, although it presents significant side effects. The aim of the present study was to investigate the effect of oleuropein alone (20 μg/mL) and in co-treatment with ADR (50 nM), in MG-63 human osteosarcoma cells. Therefore, cellular and molecular techniques, such as MTT assay, flow cytometry, real-time Polymerase Chain Reaction (PCR), western blot and Elisa method, as well as Nuclear Magnetic Resonance (NMR) spectroscopy, were applied to unveil changes in the signal transduction pathways involved in osteosarcoma cells survival. The observed alterations in gene, protein and metabolite levels denote that OLEU not only inhibits MG-63 cells proliferation and potentiates ADR’s cytotoxicity, but also exerts its action, at least in part, through the induction of autophagy.

## 1. Introduction

Osteosarcoma is a mesenchymally derived, high-grade bone tumor. In the therapeutic schemes of osteosarcoma, various anti-cancer drugs such as adriamycin, cisplatin and etoposide are basically used either alone or in combination. However, despite significant improvements in patient survival and treatment of the primary tumor, some groups of patients who present with metastatic disease, particularly osteolytic bone metastases, as well as patients with tumors that recur after treatment, continue to have a poor prognosis [1,2].

Adriamycin (ADR), in particular, is an anthracycline that is used as a potent antibiotic and has been employed in anti-cancer therapy for decades. However, ADR is also well known for the toxicity it causes in normal tissues. Especially in the heart, ADR can induce an accumulative dose-dependent cardiomyopathy that ultimately leads to congestive heart failure [3]. As a result, sustained research effort has been devoted to identifying effective drugs or treatments that can additively inhibit the proliferation of cancer cells while reducing the ADR administered dose.

In the Mediterranean diet, olive oil remains the major energy donor, and possesses a valuable position in the food pyramid of Mediterranean populations. In fact, recent studies were conducted on the beneficial effects of olive oil. Olive oil is extracted mechanically from the olive drupes, the fruits of *Olea europaea* L. [4,5]. Oleuropein (OLEU), the most abundant phenolic compound in olive oil [6], exhibits several pharmacological properties, such as anti-oxidant, anti-inflammatory, anti-atherogenic and anti-cancer actions, while a number of in vitro and in vivo studies have shown the non-toxic effect of oleuropein in normal cells and tissues [7,8,9,10]. In addition, OLEU has been shown to play a cardioprotective role against acute adriamycin cardiotoxicity and has been shown to exhibit anti-ischemic and hypolipidemic properties [11]. The combinational administration of OLEU with commonly used chemotherapeutical agents is also proven to potentiate their anti-tumor dynamics [12,13]. In particular, a previous study in our lab has demonstrated that OLEU is a powerful sensitizer of the Adriamycin-mediated killing of prostate cancer cells [14]. Despite the anti-proliferative activity OLEU displays against a number of cancer types, the mechanism of its action remains largely unknown [15,16]. The nutraceutical properties of plant polyphenols have been shown in general to arise from epigenetic and biological modifications, resulting, among other things, in autophagy stimulation. Autophagy is a catabolic process crucial for cell homeostasis, whereby cells degrade and recycle their damaged proteins and organelles through the lysosomes in order to provide energy [17].

Therefore, the aim of the current study was the evaluation of OLEU, ADR and combinational OLEU–ADR treatments, and the investigation of their biological imprint on MG-63 osteosarcoma cancer cells. Furthermore, the scope of this study was the clarification of the mechanism of action of OLEU in terms of the impact on the autophagic pathways and the identification of the metabolic footprint of osteosarcoma cancer cells.

## 2. Materials and Methods

### 2.1. Oleuropein Isolation

The OLEU used in the present study was isolated from the leaves of *Olea europaea* (Oleaceae) and OLEU structure elucidation and purification was carried out using spectroscopic and spectrometric methods, as previously described [5,11]. The 1-D and 2-D NMR experiments were performed with standard pulse programs, on a 600 MHz Avance III spectrometer (Bruker GmbH, Karlsruhe, Germany). High Resolution Mass Spectrometry (HRMS and HRMS/MS) data were obtained by direct infusion method using a hybrid LTQ-Orbitrap Discovery Mass Spectrometer (Thermo Scientific, Bremen, Germany) equipped with an ESI probe, in positive mode. OLEU’s purity (>95%) is the maximum that can be obtained from a natural compound [18].

### 2.2. Cell Line

MG-63 is an osteosarcoma cell line with osteoblastic phenotype, consisting of oval-spindle-shaped cells without branching extensions, having a doubling rate of about 24 h. The MG-63 cell line (ATCC, Bethesda, MD, USA) was grown in 75 cm^2^ culture flasks at 37 °C in 5% CO_2_ atmosphere, using as a Medium Roswell Park Memorial Institute-1640 (RPMI-1640, Gibco, Gaithersburg, MD, USA) enriched with 10% fetal bovine serum (FBS, Gibco, Gaithersburg, MD, USA).

### 2.3. MTT Cytotoxicity-Proliferation Assay

MG-63 cells were plated in 96-well plates (1500 cells per well), and after 24 h, cells were treated with ADR (in a range of 3–100 nM) and/or OLEU (in a range of 3–50 μg/mL) for a total of 96 h. MTT (3-(4,5-dimethylthiazol-2-yl)-2,5-diphenyltetrazolium bromide), (Sigma Aldrich, St. Louis, MO, USA) was added at a concentration of 5 mg/mL directly to each well and remained for 4 h at 37 °C. After the end of the aforementioned time, the medium was aspirated, and the blue MTT formazan precipitate was dissolved in dimethyl sulfoxide (DMSO). A Powerwave microplate spectrophotometer (Biotek Instruments, Inc., Winooski, VT, USA) was used for the determination of the absorbance at a wavelength of 540 nm and the results are presented as the percent of Optical Density (OD) in the treated cells versus the controls [14,19].

### 2.4. Flow Cytometry

MG-63 cells were incubated with different treatment schemes (ADR at 50 nM and OLEU at 20 μg/mL, alone and in co-treatment) at 3 different time points (24, 48 and 72 h). Cells were then washed with Phosphate Buffer Saline (PBS), fixed with 50% *v*/*v* ethanol and stained with an RNAse-containing propidium iodide (PI) solution (50 μg/mL). A BD Accuri C6 flow cytometer (BD Biosciences, Franklin Lakes, NJ, USA) and the corresponding BD CSampler software (v1.0.264.21) was employed for the DNA content analysis. Non-apoptotic events were used to calculate the percentage of cells distributed in each phase. The detailed protocol has been previously described [20].

### 2.5. Quantitative Real-Time Polymerase Chain Reaction (qRT-PCR)

Cells were seeded in 25 cm^2^ culture flasks and treated with ADR (50 nM) or/and OLEU (20 μg/mL) for 48 h and then rinsed with cold PBS solution. Total RNA content was extracted using Nucleozol reagent (Macherey-Nagel, Düren, Germany) and quantified by Nanodrop (Fischer, Waltham, MA, USA). For first-strand cDNA synthesis, 1 μg RNA was reverse transcribed with a Prime Script RT Reagent kit (Takara, Mountain View, CA, USA). Then, cDNAs of autophagy-related genes *LC3A*, *ULK1*, *AMBRA1* and *BniP3L* were subjected to quantitative real-time PCR analysis using PowerUPSYBR Green Master Mix (Fischer, Waltham, MA, USA). The sequences of the primers used in the current study and the detailed real-time PCR protocol have been previously reported [20,21,22].

### 2.6. Western Blotting

Cells were seeded into 25 cm^2^ culture flasks and treated with ADR (50 nM) or/and OLEU (20 μg/mL) for 48 h. Total protein was extracted from cells and a western blot for LC3-I (microtubule-associated protein 1A/1B-light chain 3) and LC3-II was performed as previously described [20].

### 2.7. ELISA Method

NBR1 (neighbour of BRCA1 gene 1 protein) and p62 (ubiquitin-binding protein p62) ELISA kits (Enzo, New York, NY, USA) were used for the measurement of NBR1 and p62 protein concentrations, respectively, following the IFUs (Instructions For Use) and using a Powerwave microplate spectrophotometer (Biotek Instruments, Inc. Winooski, VT, USA), at a wavelength of 450 nm [20].

### 2.8. Metabolites Extraction and Preparation of NMR (Nuclear Magnetic Resonance) Samples

Cells were seeded into 75 cm^2^ culture flasks and treated with ADR (50 nM) or/and OLEU (20 μg/mL) for 48 h. At 48 h medium was removed, cells were washed twice with PBS and were collected using a cell scrapper (Sigma Aldrich, St. Louis, MO, USA). Cells were counted in every step by Trypan blue, the culture timeline protocols for plating and cell collection were strictly respected and all the treatments were performed over the same time in all flask samples, in order to retrieve repetitive and reliable results. Then, cells were centrifuged in a 15 mL tube for 5 min at 1550× *g* and finally the supernatants were removed. Cell pellets (10^7^ cells/NMR sample) were stored at −80 °C till the extraction procedure. A previously described [23], three-solvent protocol (methanol–chloroform–water) (*n* = 6 repetitions/group) was applied. Ice-cold methanol and chloroform were added to the frozen cell pellets in a ratio of 2:1 (*v*/*v*; 250 μL/cell pellet). The cell pellet–solvent mixture was sonicated for 5 to 10 min. Chloroform and distilled water were added to the samples in a ratio of 1:1 (250 μL/cell pellet), and the samples were vortexed for 1 min to form an emulsion, left on ice for 10 min, and centrifuged at 14,000× *g* for 20 min at 4 °C. The extraction procedure was repeated twice, the collected fractions were merged, and the solvents were evaporated to dry with a centrifugal evaporator. The aqueous cell extracts were reconstituted in 700 μL of 99.9% deuterium oxide (D_2_O) at pH 7.4 phosphate buffered (150 mM) containing 0.01% 1,1,2,2-tetradeutero-3-trimethylsilylpropionic acid (TSP) as internal standard and 0.2 mM sodium azide (NaN_3_) for microbial growth inhibition [20].

### 2.9. NMR Profiling

All NMR spectra were recorded on a 600 MHz Avance III spectrometer (Bruker GmbH, Karlsruhe, Germany) equipped with a z-gradient inverse detection 5 mm probe, a B-ACS-60 sample changer controlled by ICON NMR v5.0.7 automation software (Bruker Biospin, GmbH, Karlsruhe, Germany), and a Bruker Control Unit (BCU) for temperature control. The NMR profiling (pulse sequences of choice and parameters set) for cell extracts samples has been previously described in detail by our research group [14].

### 2.10. Statistical Analysis

The biological assays data were compared using analysis of variance (one-way ANOVA). Statistical analysis was performed in triplicate determinations at *p* < 0.05.

In the case of the NMR, the spectra were segmented into buckets of 0.005 ppm width using the software package AMIX Statistics v3.9.14 (Analysis of MIXtures, Bruker BioSpin GmbH, Karlsruhe, Germany), and bucket integrals were imported into data sheets. The residual water trace (5.05–4.70 ppm) and HEPES (4-(2-hydroxyethyl)-1-piperazineethanesulfonic acid) resonances present in cell extracts (3.90–3.84, 3.19–3.05, and 3.00–2.78 ppm) were excluded. Multivariate statistical analysis was used in order to investigate the alterations in the intracellular metabolome of the different groups. The analysis was performed with SIMCA v14.1 (Umetrics, Malmö, Sweden) using both unsupervised (principal component analysis, PCA) and supervised (projection to latent structures discriminant analysis, (PLS-DA) and orthogonal PLS-DA, (OPLS-DA)) methods. The permutation test with 100 random changes, supplied by SIMCA software, was used to check for validity in the generated models. The assigned metabolites were also subjected to univariate analysis using GraphPad Prism v6.01 (GraphPad Software, Inc. San Diego, CA, USA). The Kolmogorov–Smirnov test was used to check normality, and all the three available methods (ROUT, Grubbs’ and iterative Grubbs’) for outliers’ identification were used. Statistical significance was assessed with both ordinary one-way analysis of variance (ANOVA) with Tukey’s correction for multiple comparisons and Students’ *t*-test (a *p*-value <0.05 was considered for significance) [20].

## 3. Results

### 3.1. Cytotoxicity of ADR or/and OLEU

**Figure 1:**
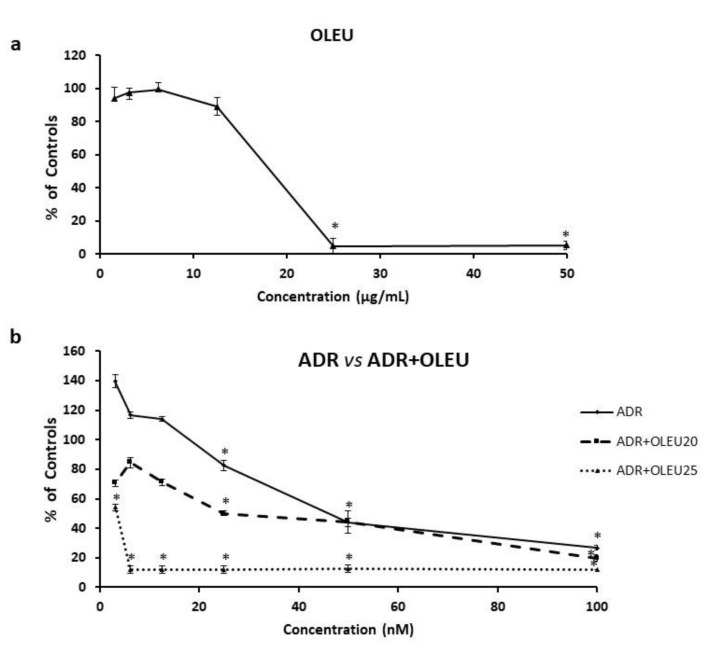
Dose–response curves for the continuous administration of (**a**) OLEU (Oleuropein) alone (3–50 μg/mL) and (**b**) ADR (Adriamycin) alone and in co-treatment schemes with either 20 μg/mL OLEU or 25 μg/mL OLEU on MG-63 cells. * *p* < 0.05 compared to control cells.

The continuous exposure of MG-63 cells to OLEU (3–50 μg/mL) produced a dose-dependent inhibition of cell proliferation, with an IC50 (half maximal inhibitory concentration) value of 22 μg/mL ± 3.6 (Figure 1a). The continuous exposure to ADR (3–100 nM) also caused a dose-dependent inhibition in the growth of MG-63 cells, with an IC50 value of 51.6 nM ± 8.4 (Figure 1b). The co-treatment with ADR (3–100 nM) and 20 μg/mL of OLEU led to an additive inhibition of cell proliferation even at very low doses of ADR (3–12.5 nM), whereas the co-treatment of ADR (3–100 nM) with 25 μg/mL of OLEU seems to be highly toxic for the cells’ viability (Figure 1b). The results mentioned in Figure 1 are expressed as percentages of controls and the experiments were performed in triplicate (*p* < 0.05). Our preliminary experiments in human fibroblasts WI-38 confirmed the lack of toxicity of OLEU in the doses tested herein (IC50 > 200 μg/mL).

### 3.2. Alterations in Cell Cycle Distribution

**Figure 2:**
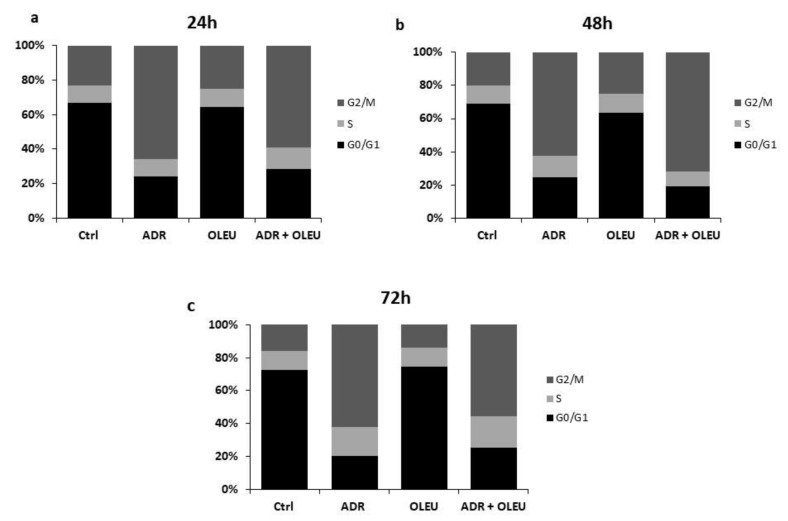
Flow cytometry graphs showing DNA content in MG-63 cells, after (**a**) 24 h, (**b**) 48 h and (**c**) 72 h of exposure to ADR (Adriamycin) and OLEU (Oleuropein), alone and in co-treatment, in comparison to control cells (Ctrl). G0: gap0, G1: gap1, G2: gap2, M: mitosis, S: synthesis.

The treatment of MG-63 cells with 20 μg/mL of OLEU for 24 h, 48 h and 72 h produced a limited increase in the cell distribution in the G0/G1 (Gap 0/ Gap 1) phase, while treatment with 50 nΜ of ADR showed the expected G2/M (Gap 2/ Mitosis) phase blockade. The addition of OLEU to the treatment with ADR did not alter the effect exhibited by ADR alone (Table 1, Figure 2 and Supplementary Figure S2).

### 3.3. Autophagy Related Genes’ Expression

Three of the autophagy-related genes tested, i.e., AMBRA1 (Activating Molecule In BECN1-Regulated Autophagy Protein 1), ULK1 (Unc-51 Like Autophagy Activating Kinase 1) and BNiP3L (BCL2 Interacting Protein 3 Like), shared a very similar expression pattern, being enhanced, statistically significantly, by ADR alone, and even more pronounced by OLEU alone, whereas the concomitant treatment ADR+OLEU appeared to function as a neutralization reaction, which almost restored gene expression to control levels.

**Figure 3:**
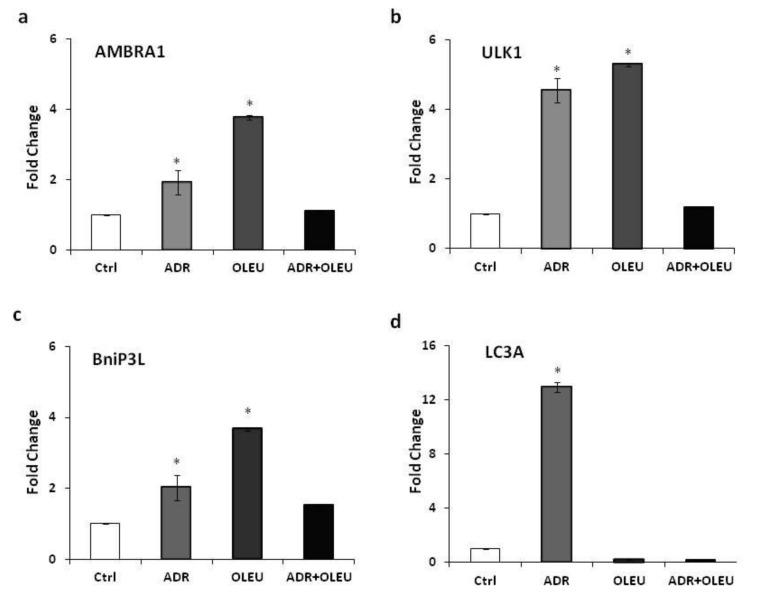
Expression levels of autophagy-related genes (**a**) AMBRA1, (**b**) ULK1, (**c**) BNiP3L, (**d**) LC3A, in MG-63 cells after ADR, OLEU, ADR + OLEU treatments, as determined by qRT-PCR. mRNA ratios are normalized to ATPase. * *p* < 0.05 compared to control cells. AMBRA1: Activating Molecule in BECN1-Regulated Autophagy Protein 1; ULK1: Unc-51 Like Au Table 1. BNiP3L; BCL2 Interacting Protein 3 Like; LC3A: microtubule-associated protein 1A light chain 3; white: control, grey: ADR (Adriamycin), dark grey: OLEU (Oleuropein), black: ADR+OLEU.

More specifically, the mRNA level of AMBRA1 was elevated (1.93 ± 0.23, fold change) after ADR treatment, and OLEU caused a more evident effect on the AMBRA1 mRNA (3.76 ± 0.02, fold change), whereas the concomitant treatment of ADR+OLEU showed no difference compared to the control (Figure 3a). In addition, ADR and OLEU significantly upregulated the mRNA expression of ULK1 (4.73 ± 0.23 and 5.42 ± 0.02), in comparison to the ADR+OLEU treatment (Figure 3b). Furthermore, ADR and OLEU alone augmented the mRNA level of BNiP3L (2.00 ± 0.23 and 3.7 ± 0.02, fold change, respectively), while the ADR+OLEU co-treatment resulted in a 1.5-fold increase in BNiP3L mRNA level (1.51 ± 0.004) (Figure 3c). Interestingly, the expression of LC3A mRNA was highly enhanced after ADR treatment, yet LC3A was almost completely suppressed after OLEU and ADR+OLEU concomitant treatment (Figure 3d). The mRNA levels of all autophagy-related genes tested were evaluated in comparison to ATPase, which served as a reference gene.

### 3.4. Expression of LC3 (Microtubule-Associated Protein 1A/1B-Light Chain 3) Proteins

**Figure 4:**
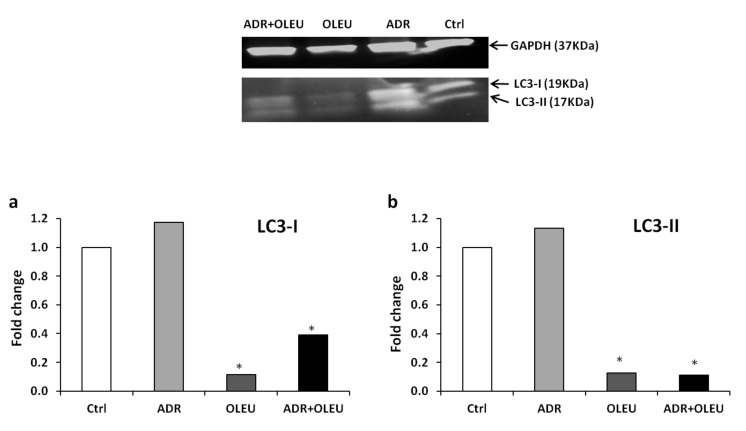
Representative western blot densitometry of the expression of the unconjugated (LC3-I) and conjugated forms (LC3-II) of LC3 in MG-63 cells, after ADR (Adriamycin), OLEU (Oleuropein), ADR + OLEU treatments, normalized against GAPDH. Protein ratios of LC3-I (**a**) and LC3-II (**b**) were used to quantify fold change relative to control. * *p* < 0.05 compared to control cells. LC3: microtubule-associated protein 1A/1B-light chain 3; GAPDH: Glyceraldehyde 3-phosphate dehydrogenase.

The expression rate of LC3B proteins was examined by western blot in order to estimate the induction of autophagy at the protein level. ADR treatment alone slightly enhanced, while OLEU and ADR+OLEU treatments significantly diminished, the expression of LC3B-I (Figure 4a) and LC3B-II (Figure 4b). Thus, the LC3B-II/LC3B-I ratio was remarkably low in the case of the ADR+OLEU concomitant treatment. Overall, the expression of *LC3B* proteins was strongly suppressed after the OLEU and ADR+OLEU treatments.

### 3.5. NBR1 (Neighbour of BRCA1 Gene 1 Protein) and p62 (Ubiquitin-Binding Protein p62) Expression

**Figure 5:**
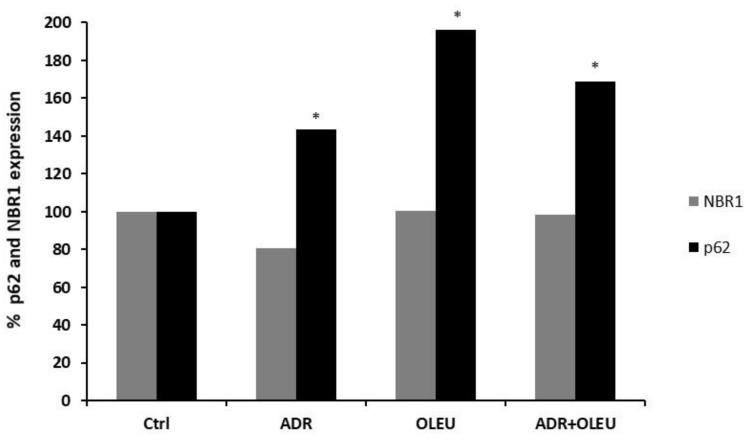
Representative graph of expression levels of NBR1 and p62 autophagy-related proteins in MG-63 cells after ADR (Adriamycin), OLEU (Oleuropein), ADR + OLEU treatments. * *p* < 0.05 compared to control cells. NBR1: neighbour of BRCA1 gene 1 protein; p62: ubiquitin-binding protein p62.

ADR treatment reduced NBR1 expression (0.8-fold change); however, OLEU and ADR+OLEU treatments did not significantly affect NBR1′s expression levels. On the contrary, all treatments enhanced p62 levels (1.4-fold change for ADR, 1.7-fold change for ADR+OLEU), with OLEU treatment alone being the most potent (twofold change) (Figure 5). Protein ratios were used to quantify fold change relative to the control in representative graphs.

### 3.6. Metabolic Profiling

**Figure 6:**
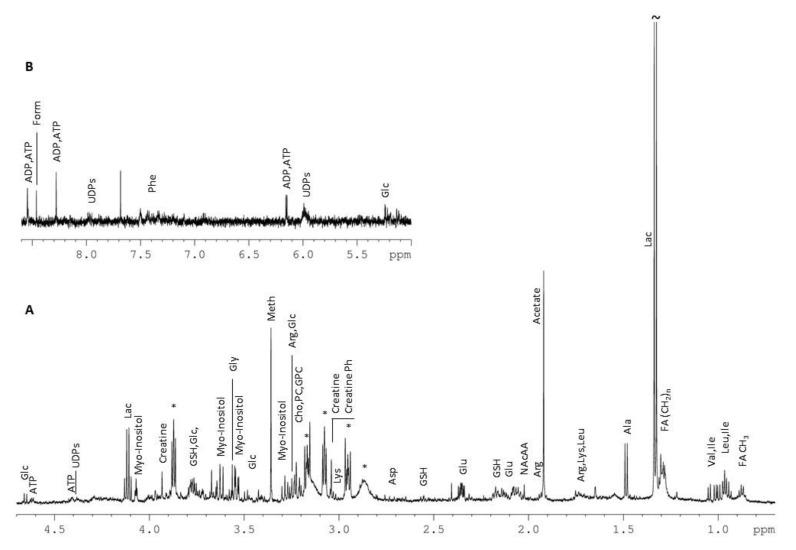
^1^H 1D NMR (One-dimensional Nuclear Magnetic Resonance) spectrum of the aqueous extracts of MG-63 cells treated with ADR (Adriamycin) and OLEU (Oleuropein) (**A**): 4.70–0.70 ppm and (**B**): 8.60–5.00 ppm). The spectrum intensity in panel B is four times maximized, compared to panel A. Asterisks point to HEPES (4-(2-hydroxyethyl)-1-piperazineethanesulfonic acid) resonance peaks. "~" used for peaks whose intensity is not fully displayed. * *p* < 0.05 compared to control cells. The spectrum intensity in panel B is four times maximized, compared to panel A. ADP: Adenosine diphosphate, ATP: Adenosine triphosphate, Form: Formate, UDPs: Uridine diphosphates, Phe: Phenylalanine, Glc: Glucose, Lac: Lactate, GSH: glutathione, Gly: Glycine, Meth: Methanol, Arg: Arginine, Cho: Choline, PC: O-Phosphocholine, GPC: Glycerophosphocholine, Lys: Lysine, Asp: Aspartate, Glu: Glutamate, NAcAA: N-Acetylated Amino Acids, Ala: Alanine, Lac: Lactate, Val: Valine, Ile: Isoleucine, Leu: Leucine, FA: fatty acids.

A representative ^1^H 1D NMR (One-dimensional Nuclear Magnetic Resonance) spectrum of an ADR+OLEU treated sample, along with the metabolites’ assignment, is shown in Figure 6.

A total of 36 metabolites comprising amino acids, cholines, nucleotides derivatives and organic acids were recognized, and are listed in detail in Table 2.

**Figure 7:**
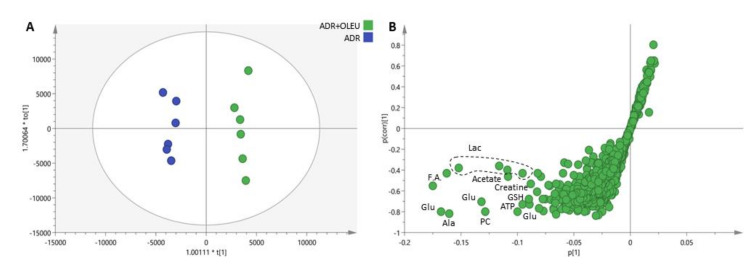
(**A**) OPLS-DA scores plot (R2Xcum = 0.76, Q2cum = 0.31) and (**B**) S-plot of pareto scaled ^1^H NMR data of the aqueous extracts of MG-63 cells treated with ADR (50 nM) and with the mixture of ADR (Adriamycin) and OLEU (Oleuropein) (50 nM and 20 μg/mL, respectively). Glu: Glutamate, Ala: Alanine, PC: O-Phosphocholine, ATP: Adenosine triphosphate, GSH: glutathione, F.A.: Fatty acids, Lac: Lactate; OPLS-DA: Orthogonal Partial Least Squares Discriminant Analysis; R2Xcum: cumulative explained variation; Q2cum: predicted variation.

The spectroscopic data were transformed to numerical values (in the form of peak-bucket integrals) and were statistically analyzed using both multivariate and univariate approaches in order to unveil changes in cell metabolism related to the different treatments applied. Both unsupervised (PCA) and supervised (PLS-DA) analysis did not reveal any clustering tendency of the examined groups, suggesting that only subtle differences were reflected on the metabolite level. In order to describe the impact of OLEU on the MG-63 cells metabolome when co-administrated with ADR, relevant comparisons between groups have been performed. Both PLS-DA and OPLS-DA (scores plotted in Figure 7A and S-plot shown in Figure 7B) point out alanine, glutamate, glutathione, lactate, acetate, Adenosine triphosphate (ATP) and phosphocholine as the metabolites that discriminate the two groups, and which are upregulated in the ADR treatment.

The statistical characteristics of the variables have also been described by means of univariate analysis. In Supplementary Table S1 the z-score values have been calculated taking the control group as the reference. The visual inspection of Supplementary Table S1 supports the results of the multivariate analysis concerning the effect of the different treatments on the metabolites’ expression. Except glycerophosphocholine, which experiences a reduction in all groups, statistically significant changes occur only in the ADR+OLEU treatment, and more precisely glutamate, glutathione, phosphocreatine, ATP, UDPs and phosphocholine are downregulated compared to control values. In Supplementary Figure S1, the peak intensities of the assigned metabolites are presented as box plots and the statistical significance (*t*-test and/or ANOVA) is marked.

Since most of the assigned metabolites present a reduction compared to the control group, except histidine and choline metabolites ratios, the fold changes were calculated as an alternative approach in order to rule out the possibility of having sample concentration effects and to investigate thoroughly the differences between ADR+OLEU co-administration and ADR treatment. The results are shown as a heat map presentation in Supplementary Table S2, and reveal an increase in choline, histidine and phenylalanine ratios, while a reduction is observed for the ratios of glutamate, N-acetyl amino acids (NAcAA), glutathione (GSH), creatine, creatine phosphate, ATP, glycerophosphocholine and phosphocholine in agreement with the results of the aforementioned statistical analyses. Finally, choline ratios (O-Phosphocholine/Choline (PC/Cho), Glycerophosphocholine/Choline (GPC/Cho), and O-Phosphocholine/Glycerophosphocholine (PC/GPC)), particularly sensitive markers of MG-63 [24,25], have been calculated for all groups, and are summarized in Supplementary Table S3. ADR administration induces a relative reduction of PC/Cho and GPC/Cho compared to control values. This reduction is further augmented upon OLEU co-administration and becomes statistically significant compared to both Ctrl and ADR treatment. The PC/GPC ratio is not affected by both ADR and ADR+OLEU treatment (Supplementary Table S3).

## 4. Discussion

Adriamycin is an antineoplastic agent that has a large therapeutic range, and it has been therefore widely used as a chemotherapeutic for more than 30 years, both on its own and in co-treatment schemes with other antineoplastic agents in the treatment of breast, ovarian, pancreatic, osteosarcoma, and a number of other cancer types [20,26]. Despite its potential against cancer cell viability, adriamycin does not exhibit selectivity in its action, resulting in its limitation in use due to undesirable side effects, including cardiotoxicity and chemoresistance [27,28]. Previous work carried out in our laboratory has highlighted the beneficial effect of ADR co-treatment with the natural olive-derived product, oleuropein, against PC-3 prostate cancer cells, and the finding that the combinational scheme enhances the cytotoxic effect of ADR by interfering with the autophagic potential of the cells [14].

In the present study, the treatment of MG-63 cells with OLEU alone inhibited cell proliferation in a dose-dependent manner. Additionally, the co-treatment with ADR+OLEU enhanced the inhibition of cell proliferation in significantly lower ADR doses than the ADR treatment alone. The anti-cancer effect of OLEU against osteosarcoma cell lines was previously described [29], whereas the additive effect of ADR and OLEU has been previously shown in breast cancer in in vitro and in vivo models [26,30].

In order to elucidate the molecular mechanism underlying this ADR+OLEU effect in osteosarcoma, cell cycle analysis was conducted. The observed cell cycle changes after ADR treatment coincide with previous studies, which also highlight the proliferation-related genes implicated, such as p53 and p21 (WAF1/CIP1) [31,32,33,34]. On the other hand, cell cycle distribution is not altered, nor is apoptosis induced, following OLEU treatment, whereas the ADR+OLEU co-treatment resembles the ADR alone, causing the expected G2/M cell cycle arrest. Taking into consideration this result, it was suggested that a mechanism other than apoptosis, potentially autophagy, is implicated.

Autophagy is activated by cells in extreme physiological conditions, such as nutrient deprivation, in order to prolong cell survival by the self-digestion of cytoplasmic proteins and organelles [35]. Multiple genes, such as AMBRA1, ULK1, BniP3L and LC3, are involved in the autophagic machinery. Even though there are distinct LC3A and LC3B autophagosomes, LC3A and LC3B are equally used to access the autophagic activity [36]. In the present study, the expression of autophagy-related genes after ADR treatment suggests the induction of autophagy, which serves as a survival mechanism in MG-63 cells, as it is elsewhere reported [37]. Furthermore, the gene expression profile of MG-63 cells revealed that OLEU treatment alone strongly enhanced the expression of ULK1, AMBRA1 and BniP3L mRNAs, whereas LC-3 expression was greatly suppressed, supporting the notion of the OLEU-mediated induction of autophagy and probably mitophagy, in a higher rate compared to ADR, coinciding with the findings of previous studies in different cell lines [38,39,40]. In the case of the ADR+OLEU concomitant treatment, the additive effect of OLEU-induced autophagy to the established ADR-induced protective autophagy results in the dismantling of the autophagic machinery.

Furthermore, the expression levels of p62 protein (autophagic flux marker) are also increased following the treatments, with OLEU administration showing the most pronounced effect. Usually, the levels of p62 protein are diminished upon autophagolysome degradation, and the accumulation of p62 protein suggests that the autophagic process could be either defective or in an early state of autophagosome–lysosome fusion [41,42]. On the contrary, there are studies which suggest that the accumulation of the p62 protein is consistent with stress-mediated autophagy [43]. Taken together, in the autophagy–lysosome system, the proteins LC3, p62 and BniP3L are critical for membrane commitment, cargo delivery and the selective removal of damaged mitochondria, respectively. To elicit their functions, these proteins are entrapped in the autophagosome when the vesicle is formed, and therefore are destroyed upon the fusion of autophagosome with lysosome.

The biological effect of OLEU and ADR+OLEU against MG-63 osteosarcoma cells was further investigated on the metabolite level because signaling pathways are of crucial importance in cancer, and are greatly affected by metabolite alterations. Both multi- and univariate analyses did not reveal any considerable difference induced on the metabolome level upon ADR administration, except glycerophosphocholine’s statistically significant reduction. However, when the natural product OLEU is co-administrated with ADR, the impact is more pronounced, and statistically significant reduction is detected for glutamate, glutathione, phosphocreatine, ATP, Uridine Diphosphates (UDPs) and phosphocholine, while glycerophosphocholine is further decreased. The metabolite ratios comparisons confirmed these observations and indicated a reduction in PC/Cho and GPC/Cho in the ADR treatment compared to control values, which become more prominent in ADR+OLEU co-administration. The alterations in these metabolites further support the implication of autophagy in the underlying molecular mechanism. More specifically, ATP is required to provide energy for all cellular processes, and cancer cells promote autophagy in order to overcome the energy shortage from the glycolysis pathway, the major energy source, by maintaining mitochondrial activity to produce the ATP necessary for survival [44]. The decrease in its hydrolysis product, ADP, is consistent with the strong inhibition of cell proliferation caused after ADR+OLEU treatment. Furthermore, the downregulation of glutamate further supports the implication of the autophagic pathways; cancer cells show an increased intake of glutamine undergoing glutaminolysis, which is strongly correlated with the nutrient-sensitive kinase, mammalian target of rapamycin complex 1 (mTORC1), which regulates protein and lipid synthesis, gene transcription and autophagy [45].

Additionally, the reduction in glutathione would be expected given the elevated oxidative status in which cancer cells survive. Choline metabolism is also one of the most consistent cancer hallmarks; the elevation of choline metabolites is closely related to cancer cell proliferation, and in the last decades has emerged as a promising target for cancer therapy [46]. Τhe major importance of choline metabolism has been previously reported in MG-63 cells, following exposure to ADR at a dose of 3 µM for three different time courses, i.e., 12, 24 and 48 h were investigated by means of ^1^H HRMAS NMR spectroscopy [25]; herein, ADR treatment was found to induce enhanced membrane degradation, accompanied by decreased phosphatidylcholine (PTC) synthesis and the apparent inhibition of de novo lipid synthesis [24,25]. Although in the current work ADR impact was exploited at a 60-fold lower concentration (50 nM), the results, regarding the observed alteration in choline metabolism, coincide; besides this, the decrease in phosphocholine is strongly correlated with the induction of autophagy in cancer cells [47,48]. Finally, there is increasing evidence that creatine phosphate and the creatine pathway are directly linked to metabolic stress, as they play a pivotal role in coordinating stress response in the tumor microenvironment [49].

Taken together, after ADR treatment, the replenishment of the cytoplasm with the metabolites mentioned, combined with the changes observed in lipid synthesis and the expression of autophagy-related genes and proteins, implies a synchronized autophagic activation to fuel the cells with the required energy to achieve survival. The results after OLEU treatment also indicate the induction of autophagy and, potentially, mitophagy at higher rates compared to ADR. Furthermore, a recent study in MCF-7 breast cancer cells revealed the induction of autophagy as a protective cell mechanism after OLEU treatment [50]. Overall, our data reveal that the addition of OLEU to ADR further enhances the ADR-induced protective autophagy in MG-63 cells, provoking an exorbitant metabolic imbalance and the cellular dismantling of the autophagic machinery, which eventually leads to cell death.

## 5. Conclusions

In conclusion, OLEU has anti-proliferative properties against MG-63 cells, and enhances ADR cytotoxicity by interfering with the ADR-induced autophagic potential of the cells. Since OLEU is a natural and edible bioactive product with no side effects, it could serve as a potential candidate for the design of a more effective adjuvant treatment for osteosarcoma patients.

## Figures and Tables

**Table 1:** Cell cycle phase distribution after 24, 48, 72 h (%) of exposure to ADR (Adriamycin) and OLEU (Oleuropein), alone and in co-treatment, in comparison to control cells (Ctrl).

	Cell Cycle Phase Distribution	G0/G1	S	G2/Μ
Treatments				
		24 h		
Ctrl		66.9	10.2	22.9
ADR		24	10.1	65.9
OLEU		64.6	10.5	24.9
ADR + OLEU		28.3	12.6	59.1
		48 h		
Ctrl		69	11.2	19.8
ADR		24.6	12.8	62.6
OLEU		63.3	11.9	24.8
ADR + OLEU		19.1	9.2	71.7
		72 h		
Ctrl		72.3	11.7	16
ADR		20.1	17.7	62.2
OLEU		74.6	11.4	14
ADR + OLEU		25.4	19.2	55.4

Ctrl: control cells; ADR: adriamycin; OLEU: Oleuropein; G0/G1: Gap 0/ Gap 1; G2/Μ: Gap 2/ Mitosis; S: synthesis.

**Table 2:** ^1^H chemical shifts (ppm) of metabolites identified in the ^1^H NMR spectra of aqueous extracts of untreated MG-63 cells and those treated with ADR (50 nM), OLEU (20 µg/mL) and ADR+OLEU (50 nM + 20 µg/mL) for 48 h.

No	Metabolites	Chemical Shift (ppm) (Multiplicity)
	**Amino Acids and Derivatives**	
1	Alanine (Ala)	3.79 (q); 1.48 (d)
2	Arginine (Arg)	3.25 (t); 1.94 (m)
3	Aspartate (Asp)	2.81 (dd); 2.69 (dd)
4	Glutamate (Glu)	3.76 (q); 2.35 (m); 2.13 (m); 2.06 (m)
5	Glycine (Gly)	3.56 (s)
6	Histidine (His)	7.87; 7.10
7	Isoleucine (Ile)	1.01 (d); 0.94 (t)
8	Leucine (Leu)	0.97 (d); 0.96 (d)
9	Lysine (Lys)	3.03 (t); 1.74 (m)
10	Phenylalanine (Phe)	7.43 (m); 7.38 (m); 7.34 (d)
11	Threonine (Thr)	1.335 (d)
12	Tyrosine (Tyr)	7.20 (d); 6.90 (d)
13	Valine (Val)	1.05 (d); 1.00 (d)
14	Creatine	3.93 (s); 3.04 (s)
15	Creatine Phosphate	3.045 (s)
16	Glutathione reduced (GSH)	3.77 (m); 2.56 (m); 2.17 (m)
17	N-Acetylated Amino Acids (NAcAA)	2.02 (s)
18	Taurine	3.42 (t); 3.25 (t)
	**Organic Acids**	
19	Acetate	1.92 (s)
20	Formate (Form)	8.46 (s)
21	Lactate (Lac)	4.11 (q); 1.33 (d)
22	Succinate (Succ)	2.41 (s)
	**Nucleotides and Derivatives**	
23	Adenosine diphosphate (ADP)	8.535 (s)
24	Adenosine triphosphate (ATP)	8.545 (s); 8.28 (s); 6.15 (d); 4.62 (m); 4.41 (t)
25	Uridine Diphosphates (UDPs)	7.98 (d); 5.98 (m)
26	Uridine Diphosphate (UDP-GlucNAc)	7.95 (d); 5.98 (m); 2.08 (s)
	**Choline and Derivatives**	
27	Choline (Cho)	3.21 (s)
28	O-Phosphocholine (PC)	3.225 (s)
29	Glycerophosphocholine (GPC)	3.235 (s)
	**Lipid Fatty Acids**	
30	CH_3_	0.88
31	(CH_2_)_n_	1.29
32	CH_2_CH_2_CO	1.55
	**Other**	
33	Glucose (Glc)	5.24 (d); 4.65 (d); 3.90 (dd); 3.84 (m); 3.73 (m); 3.50 (t); 3.47 (m); 3.41 (m); 3.25 (dd)
34	Myo-Inositol	4.07 (t); 3.63 (t); 3.54 (dd); 3.29 (t)
35	Glycerol	3.65 (dd); 3.56 (dd)
36	Methanol (Meth)	3.36 (s)

## Data Availability

Not applicable.

## Supplementary Materials

The following are available online at https://www.mdpi.com/2072-6643/13/2/354/s1. Figure S1: Box plots demonstrating the statistically significant relative concentration of specific metabolites between the different treatments (Ctrl, ADR, OLEU, ADR+OLEU). Figure S2: Flow cytometry graphs showing DNA content in MG-63 cells, after exposure to ADR (Adriamycin) and OLEU (Oleuropein), alone and in co-treatment, as well as in control cells (Ctrl). Table S1: Z−score values of the metabolites observed in the 1H 1D NMR spectra of the aqueous extracts of MG−63 cells treated with ADR (50 nM), OLEU (20 µg/mL) and ADR+OLEU (50 nM+20 µg/mL) compared to untreated cells (Ctrl). Table S2: Heat map presentation of fold change values of the assigned metabolites ratios (nominator in column and denominator in row) between ADR+OLEU and ADR treatment. Table S3: Choline metabolites ratios of untreated MG-63 cells (Ctrl) and exposed to ADR. OLEU. and ADR+OLEU for 48 h. For ratios calculation mean values of the corresponding integrals from the 1H NMR profiles were used.
