# Supplementary material for: Assessment of the Nutraceutical Effects of Oleuropein and the Cytotoxic Effects of Adriamycin, When Administered Alone and in Combination, in MG-63 Human Osteosarcoma Cells
Source: Nutrients. 2021 Jan 25;13(2):354. doi: 10.3390/nu13020354 (PMC7911555; doi:10.3390/nu13020354)
Supplement: Supplementary file 1 [file nutrients-13-00354-s001.pdf]

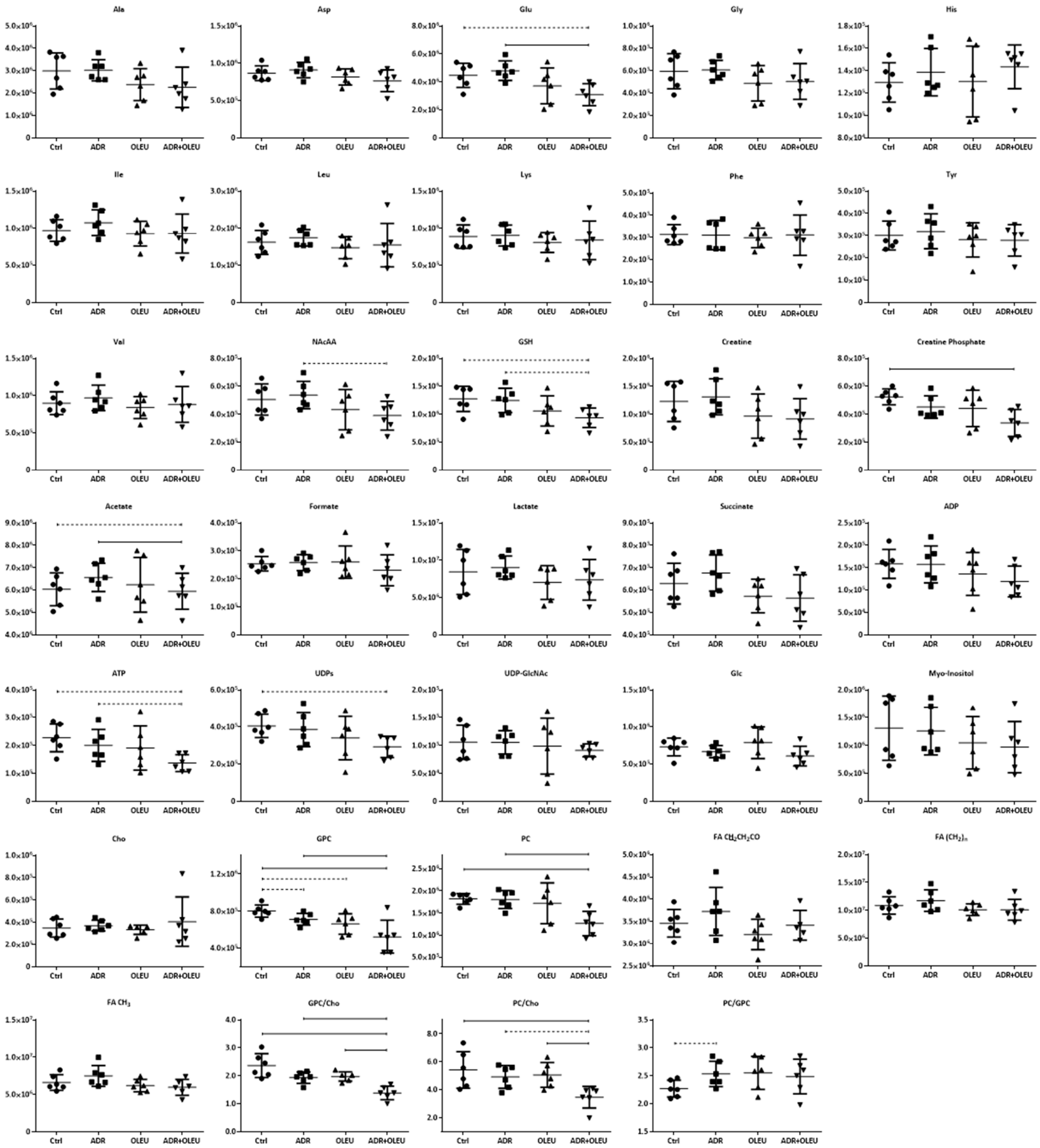

Supplementary Figure S1. Box plots demonstrating the statistically significant relative concentration of specific metabolites between the different treatments (Ctrl, ADR, OLEU, ADR+OLEU). ADP: Adenosine diphosphate, ATP: Adenosine triphosphate, Form: Formate, UDPs: Uridine diphosphates, UDP GlcNAc: Uridine diphosphate N-acetylglucosamine, Phe: Phenylalanine, Tyr: Tyrosine, Glc: Glucose, Lac: Lactate, GSH: glutathione, Gly: Glycine, His: Histidine, Arg: Arginine, Cho: Choline, PC: O-Phosphocholine, GPC: Glycerophosphocholine, Lys: Lysine, Asp: Aspartate, Glu: Glutamate, NAcAA: N-Acetylated Amino Acids, Ala: Alanine, Lac: Lactate, Val: Valine, Ile: Isoleucine, Leu: Leucine, FA: fatty acids. Continuous line indicates the statistically significance according to one-way Analysis of Variance (one-way ANOVA) and dashed line according to t−test.

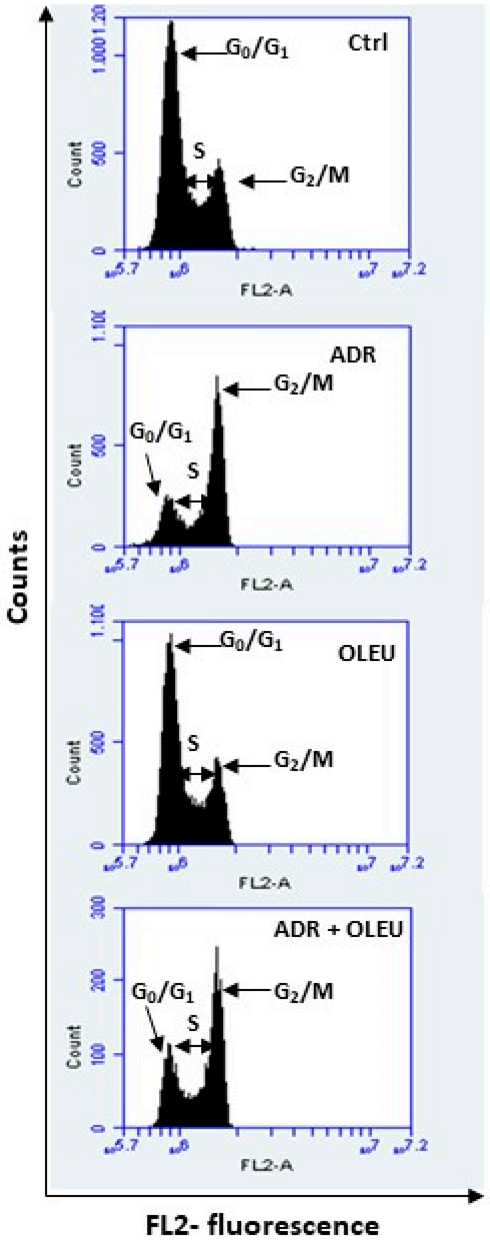

Supplementary Figure S2. Flow cytometry graphs showing DNA content in MG-63 cells, after exposure to ADR (Adriamycin) and OLEU (Oleuropein), alone and in co-treatment, as well as in control cells (Ctrl). G0: gap0, G1: gap1, G2: gap2, M: mitosis, S: synthesis.

Supplementary Table S1. Z−score values of the metabolites observed in the 1H 1D NMR spectra of the aqueous extracts of MG−63 cells treated with ADR (50 nM), OLEU (20 μg/mL) and ADR+OLEU (50 nM+20 μg/mL) compared to untreated cells (Ctrl).

Metabolites	ADR	OLEU	ADR+OLEU
Ala	0.0	−0.8	−1.0
Asp	0.5	−0.6	−1.1
Glu	0.4	−1.0	**−1.8**
Gly	0.1	−0.8	−0.6
His	0.6	0.0	0.9
Ile	0.8	−0.3	−0.3
Leu	0.4	−0.5	−0.2
Lys	0.1	−0.5	−0.3
Phe	−0.1	−0.4	−0.1
Tyr	0.3	−0.3	−0.4
Val	0.5	−0.4	−0.1
NAcAA	0.3	−0.7	−1.1
GSH	−0.1	−1.1	**−1.6**
Creatine	0.2	−0.8	−1.0
Creatine Phosphate	−1.4	−1.6	<u>**−3.6**</u>
Acetate	0.8	0.3	−0.1
Form	0.2	0.3	−1.0
Lac	0.2	−0.5	−0.4
Succ	0.6	−0.7	−0.8
ADP	0.0	−0.8	−1.3
ATP	−0.6	−0.8	**−2.0**
UDPs	−0.3	−1.1	**−1.9**
UDP−GlcNAc	0.0	−0.3	−0.5
Glc	−0.6	0.5	−1.1
Myo−Inositol	−0.1	−0.5	−0.7
Cho	0.3	−0.2	0.8
GPC	**−1.5**	**−2.3**	<u>**−4.6**</u>
PC	−0.1	−0.9	<u>**−5.0**</u>
Glycerol	0.4	−0.2	0.1
FA CH_2CH_2CO	0.9	−0.9	−0.6
FA $(CH_2)_n$	0.6	−0.5	−0.6
FA CH_3 0.88	0.9	−0.4	−0.7

Blue shades indicate the reduction of the mentioned metabolite compared to its respective level in control cells and red shades indicate the increase of its level, bold indicates the statistically significance according to t−test and underlining according to one-way Analysis of Variance (one-way ANOVA). ADP: Adenosine diphosphate. ATP: Adenosine triphosphate. Form: Formate. Succ: Succinate. UDPs: Uridine diphosphates. UDP GlcNAc: Uridine diphosphate N-acetylglucosamine. Phe: Phenylalanine. Tyr: Tyrosine. Glc: Glucose. Lac: Lactate. GSH: glutathione. Gly: Glycine. His: Histidine. Arg: Arginine. Cho: Choline. PC: O-Phosphocholine. GPC: Glycerophosphocholine. Lys: Lysine. Asp: Aspartate. Glu: Glutamate. NAcAA: N-Acetylated Amino Acids. Ala: Alanine. Lac: Lactate. Val: Valine. Ile: Isoleucine. Leu: Leucine. FA: fatty acids.

Supplementary Table S2. Heat map presentation of fold change values of the assigned metabolites ratios (nominator in column and denominator in row) between ADR+OLEU and ADR treatment.

	Ala	Asp	Glu	Gly	His	Ile	Leu	Lys	Phe	Tyr	Val	NAcAA	GSH	Creatine	Creatine Ph	Acetate	Form	Lac	Succ	ADP	ATP	UDPs	UDP-GlcNAc	Glc	Myo-Inositol	Cho	GPC	PC	Glycerol	FA $C\underline{H_2}CH_2CO$	FA $(CH_2)_n$	FA CH_3
Ala	1.0	1.1	0.9	1.1	1.4	1.2	1.2	1.2	1.3	1.2	1.2	1.0	1.0	0.9	1.0	1.2	1.2	1.1	1.1	1.0	0.9	1.0	1.2	1.2	1.0	1.5	1.0	0.9	1.3	1.2	1.1	1.1
Asp	0.9	1.0	0.8	1.0	1.2	1.0	1.1	1.1	1.2	1.0	1.1	0.9	0.9	0.8	0.9	1.1	1.1	1.0	1.0	0.9	0.8	0.9	1.0	1.1	0.9	1.3	0.9	0.8	1.1	1.1	1.0	0.9
Glu	1.2	1.3	1.0	1.3	1.6	1.3	1.4	1.4	1.6	1.4	1.4	1.1	1.2	1.1	1.2	1.4	1.4	1.3	1.3	1.2	1.1	1.2	1.3	1.4	1.2	1.7	1.1	1.1	1.5	1.4	1.3	1.2
Gly	0.9	1.0	0.8	1.0	1.2	1.0	1.1	1.1	1.2	1.0	1.1	0.9	0.9	0.8	0.9	1.1	1.1	1.0	1.0	0.9	0.8	0.9	1.0	1.1	0.9	1.3	0.9	0.8	1.1	1.1	1.0	1.0
His	0.7	0.8	0.6	0.8	1.0	0.8	0.9	0.9	1.0	0.8	0.9	0.7	0.7	0.7	0.7	0.9	0.9	0.8	0.8	0.7	0.7	0.7	0.8	0.9	0.7	1.1	0.7	0.7	0.9	0.9	0.8	0.8
Ile	0.9	1.0	0.7	1.0	1.2	1.0	1.0	1.1	1.2	1.0	1.1	0.8	0.9	0.8	0.9	1.0	1.0	0.9	1.0	0.9	0.8	0.9	1.0	1.1	0.9	1.3	0.9	0.8	1.1	1.0	1.0	0.9
Leu	0.8	0.9	0.7	0.9	1.2	1.0	1.0	1.0	1.1	1.0	1.0	0.8	0.8	0.8	0.8	1.0	1.0	0.9	0.9	0.9	0.8	0.9	1.0	1.0	0.9	1.2	0.8	0.8	1.1	1.0	1.0	0.9
Lys	0.8	0.9	0.7	0.9	1.1	0.9	1.0	1.0	1.1	0.9	1.0	0.8	0.8	0.8	0.8	1.0	1.0	0.9	0.9	0.8	0.7	0.8	0.9	1.0	0.8	1.2	0.8	0.8	1.0	1.0	0.9	0.9
Phe	0.7	0.8	0.6	0.8	1.0	0.9	0.9	0.9	1.0	0.9	0.9	0.7	0.8	0.7	0.7	0.9	0.9	0.8	0.8	0.8	0.7	0.8	0.9	0.9	0.8	1.1	0.7	0.7	1.0	0.9	0.9	0.8
Tyr	0.9	1.0	0.7	1.0	1.2	1.0	1.0	1.1	1.1	1.0	1.0	0.8	0.9	0.8	0.9	1.0	1.0	0.9	1.0	0.9	0.8	0.9	1.0	1.0	0.9	1.3	0.8	0.8	1.1	1.0	1.0	0.9
Val	0.8	0.9	0.7	0.9	1.1	0.9	1.0	1.0	1.1	1.0	1.0	0.8	0.8	0.8	0.8	1.0	1.0	0.9	0.9	0.8	0.8	0.8	0.9	1.0	0.8	1.2	0.8	0.8	1.0	1.0	0.9	0.9
NAcAA	1.0	1.2	0.9	1.1	1.4	1.2	1.2	1.3	1.4	1.2	1.3	1.0	1.0	1.0	1.0	1.2	1.2	1.1	1.1	1.0	0.9	1.0	1.2	1.3	1.1	1.5	1.0	1.0	1.3	1.2	1.2	1.1
GSH	1.0	1.1	0.9	1.1	1.4	1.2	1.2	1.2	1.3	1.2	1.2	1.0	1.0	0.9	1.0	1.2	1.2	1.1	1.1	1.0	0.9	1.0	1.2	1.2	1.0	1.5	1.0	0.9	1.3	1.2	1.1	1.1
Creatine	1.1	1.2	0.9	1.2	1.5	1.2	1.3	1.3	1.4	1.2	1.3	1.0	1.1	1.0	1.1	1.3	1.3	1.2	1.2	1.1	1.0	1.1	1.2	1.3	1.1	1.6	1.1	1.0	1.4	1.3	1.2	1.1
Creatine Ph	1.0	1.1	0.9	1.1	1.4	1.2	1.2	1.2	1.3	1.2	1.2	1.0	1.0	0.9	1.0	1.2	1.2	1.1	1.1	1.0	0.9	1.0	1.2	1.2	1.0	1.5	1.0	0.9	1.3	1.2	1.1	1.1
Acetate	0.8	0.9	0.7	0.9	1.1	1.0	1.0	1.0	1.1	1.0	1.0	0.8	0.8	0.8	0.8	1.0	1.0	0.9	0.9	0.8	0.8	0.8	1.0	1.0	0.8	1.2	0.8	0.8	1.1	1.0	0.9	0.9
Form	0.8	0.9	0.7	0.9	1.2	1.0	1.0	1.0	1.1	1.0	1.0	0.8	0.8	0.8	0.8	1.0	1.0	0.9	0.9	0.8	0.8	0.9	1.0	1.0	0.9	1.2	0.8	0.8	1.1	1.0	1.0	0.9
Lac	0.9	1.0	0.8	1.0	1.3	1.1	1.1	1.1	1.2	1.1	1.1	0.9	0.9	0.9	0.9	1.1	1.1	1.0	1.0	0.9	0.8	0.9	1.1	1.1	0.9	1.3	0.9	0.9	1.2	1.1	1.0	1.0
Succ	0.9	1.0	0.8	1.0	1.2	1.0	1.1	1.1	1.2	1.0	1.1	0.9	0.9	0.8	0.9	1.1	1.1	1.0	1.0	0.9	0.8	0.9	1.0	1.1	0.9	1.3	0.9	0.8	1.1	1.1	1.0	1.0
ADP	1.0	1.1	0.9	1.1	1.4	1.1	1.2	1.2	1.3	1.2	1.2	1.0	1.0	0.9	1.0	1.2	1.2	1.1	1.1	1.0	0.9	1.0	1.1	1.2	1.0	1.4	1.0	0.9	1.3	1.2	1.1	1.1
ATP	1.1	1.2	0.9	1.2	1.5	1.3	1.3	1.4	1.5	1.3	1.3	1.1	1.1	1.0	1.1	1.3	1.3	1.2	1.2	1.1	1.0	1.1	1.3	1.3	1.1	1.6	1.1	1.0	1.4	1.3	1.3	1.2
UDPs	1.0	1.1	0.8	1.1	1.4	1.1	1.2	1.2	1.3	1.1	1.2	1.0	1.0	0.9	1.0	1.2	1.2	1.1	1.1	1.0	0.9	1.0	1.1	1.2	1.0	1.4	1.0	0.9	1.3	1.2	1.1	1.0
UDP-GlcNAc	0.9	1.0	0.7	1.0	1.2	1.0	1.0	1.1	1.2	1.0	1.1	0.8	0.9	0.8	0.9	1.0	1.0	0.9	1.0	0.9	0.8	0.9	1.0	1.1	0.9	1.3	0.9	0.8	1.1	1.0	1.0	0.9
Glc	0.8	0.9	0.7	0.9	1.1	0.9	1.0	1.0	1.1	1.0	1.0	0.8	0.8	0.8	0.8	1.0	1.0	0.9	0.9	0.8	0.8	0.8	0.9	1.0	0.8	1.2	0.8	0.8	1.0	1.0	0.9	0.9
Myo-Inositol	1.0	1.1	0.8	1.1	1.3	1.1	1.2	1.2	1.3	1.1	1.2	0.9	1.0	0.9	1.0	1.2	1.2	1.1	1.1	1.0	0.9	1.0	1.1	1.2	1.0	1.4	1.0	0.9	1.2	1.2	1.1	1.0
Cho	0.7	0.8	0.6	0.8	0.9	0.8	0.8	0.9	0.9	0.8	0.8	0.7	0.7	0.6	0.7	0.8	0.8	0.8	0.8	0.7	0.6	0.7	0.8	0.8	0.7	1.0	0.7	0.6	0.9	0.8	0.8	0.7
GPC	1.0	1.1	0.9	1.1	1.4	1.2	1.2	1.3	1.4	1.2	1.2	1.0	1.0	0.9	1.0	1.2	1.2	1.1	1.1	1.0	0.9	1.0	1.2	1.2	1.0	1.5	1.0	1.0	1.3	1.2	1.2	1.1
PC	1.1	1.2	0.9	1.2	1.5	1.2	1.3	1.3	1.4	1.2	1.3	1.0	1.1	1.0	1.1	1.3	1.3	1.2	1.2	1.1	1.0	1.1	1.2	1.3	1.1	1.6	1.1	1.0	1.4	1.3	1.2	1.1
Glycerol	0.8	0.9	0.7	0.9	1.1	0.9	0.9	1.0	1.0	0.9	1.0	0.8	0.8	0.7	0.8	1.0	0.9	0.9	0.9	0.8	0.7	0.8	0.9	1.0	0.8	1.1	0.8	0.7	1.0	0.9	0.9	0.8
FA $C\underline{H_2}CH_2CO$	0.8	0.9	0.7	0.9	1.2	1.0	1.0	1.0	1.1	1.0	1.0	0.8	0.8	0.8	0.8	1.0	1.0	0.9	0.9	0.9	0.8	0.9	1.0	1.0	0.9	1.2	0.8	0.8	1.1	1.0	1.0	0.9
FA $(CH_2)_n$	0.9	1.0	0.8	1.0	1.2	1.0	1.0	1.1	1.2	1.0	1.1	0.8	0.9	0.8	0.9	1.1	1.0	1.0	1.0	0.9	0.8	0.9	1.0	1.1	0.9	1.3	0.9	0.8	1.1	1.0	1.0	0.9
FA CH_3	0.9	1.1	0.8	1.0	1.3	1.1	1.1	1.2	1.3	1.1	1.1	0.9	0.9	0.9	0.9	1.1	1.1	1.0	1.0	1.0	0.9	1.0	1.1	1.1	1.0	1.4	0.9	0.9	1.2	1.1	1.1	1.0

Blue shades indicate the reduction and red shades the increase of the aforementioned ratio. ADP: Adenosine diphosphate. ATP: Adenosine triphosphate. Form: Formate. Succ: Succinate. UDPs: Uridine diphosphates. UDP GlcNAc: Uridine diphosphate N-acetylglucosamine. Phe: Phenylalanine. Tyr: Tyrosine. Glc: Glucose. Lac: Lactate. GSH: glutathione. Gly: Glycine. His: Histidine. Arg: Arginine. Cho: Choline. PC: O-Phosphocholine. GPC: Glycerophosphocholine. Lys: Lysine. Asp: Aspartate. Glu: Glutamate. NAcAA: N-Acetylated Amino Acids. Ala: Alanine. Lac: Lactate. Val: Valine. Ile: Isoleucine. Leu: Leucine. FA: fatty acids.

Supplementary Table S3. Choline metabolites ratios of untreated MG-63 cells (Ctrl) and exposed to ADR. OLEU. and ADR+OLEU for 48 h. For ratios calculation mean values of the corresponding integrals from the 1H NMR profiles were used.

Treatment	PC/Cho	GPC/Cho	PC/GPC
Ctrl	5.42±1.19	2.37±0.39	2.27±0.14
ADR	4.92±0.74	1.94±0.19	2.53±0.21
OLEU	5.06±0.81	1.97±0.15	2.55±0.27
ADR+OLEU	**3.47±0.71***	**1.39±0.22***	2.48±0.28

Bold indicates the statistical significance compared to untreated cells (Ctrl) and * indicates the statistical significance ($p < 0.05$) compared to Adriamycin (ADR).
